# Decreasing body size is associated with reduced calving probability in critically endangered North Atlantic right whales

**DOI:** 10.1098/rsos.240050

**Published:** 2024-02-28

**Authors:** Enrico Pirotta, Peter L. Tyack, John W. Durban, Holly Fearnbach, Philip K. Hamilton, Catriona M. Harris, Amy R. Knowlton, Scott D. Kraus, Carolyn A. Miller, Michael J. Moore, Heather M. Pettis, Theoni Photopoulou, Rosalind M. Rolland, Robert S. Schick, Len Thomas

**Affiliations:** ^1^ Centre for Research into Ecological and Environmental Modelling, University of St Andrews, St Andrews, UK; ^2^ School of Biology, Scottish Oceans Institute, University of St Andrews, St Andrews, UK; ^3^ Southall Environmental Associates, Inc., 9099 Soquel Drive, Aptos, CA 95003, USA; ^4^ SR3, SeaLife Response, Rehabilitation and Research, Des Moines, WA, USA; ^5^ Anderson Cabot Center for Ocean Life, New England Aquarium, Boston, MA, USA; ^6^ Department of Marine Chemistry and Geochemistry, Woods Hole Oceanographic Institution, Woods Hole, MA, USA; ^7^ Department of Biology, Woods Hole Oceanographic Institution, Woods Hole, MA, USA; ^8^ Marine Geospatial Ecology Lab, Nicholas School of the Environment, Duke University, Durham, NC, USA

**Keywords:** Bayesian state-space model, body size, capital breeding, *Eubalaena glacialis*, health, length

## Abstract

Body size is key to many life-history processes, including reproduction. Across species, climate change and other stressors have caused reductions in the body size to which animals can grow, called asymptotic size, with consequences for demography. A reduction in mean asymptotic length was documented for critically endangered North Atlantic right whales, in parallel with declines in health and vital rates resulting from human activities and environmental changes. Here, we tested whether smaller body size was associated with lower reproductive output, using a state-space model for individual health, survival and reproduction that quantifies the mechanistic links between these processes. Body size (as represented by the cube of length) was strongly associated with a female's calving probability at each reproductive opportunity. This relationship explained 62% of the variation in calving among reproductive females, along with their decreasing health (20%). The effects of decreasing mean body size on reproductive performance are another concerning indication of the worsening prospects for this species and many others affected by environmental change, requiring a focus of conservation and management interventions on improving conditions that affect reproduction as well as reducing mortality.

## 1. Introduction

Across taxa, body size influences key life-history processes, resulting in variation in demographic rates among species and individuals [1,2]. In particular, several aspects of reproduction are affected by how large an animal is, including time to sexual maturity, rate of reproduction (e.g. inter-birth interval), size of offspring at birth, energy invested in the offspring, and, ultimately, an individual's reproductive success [1]. This is especially true for animals that rely on energy reserves stored prior to breeding to support reproduction (known as capital breeders [3]), where an individual's storage capacity (i.e. the reserves it can accumulate) depends on its structural size [4,5].

Recent studies have documented reductions in mean body size of many terrestrial and aquatic organisms [6]. A decline in size has been hypothesized as a common response to climate change, although the issue is debated and the ultimate mechanisms (evolution versus plasticity) are unclear. Proximately, smaller sizes have been ascribed to changes in growth resulting from impaired nutrition linked with reduced availability or quality of food resources [6]. Such changes can affect the maximum size individuals can reach (i.e. their asymptotic size). In some populations, reductions in mean individual size emerge from size-selective removal of larger individuals (e.g. [7]) or from anthropogenic stressors that hinder growth (e.g. [8]). In general, decreasing body size has been proposed as an early warning of impending population collapse [9].

Alterations in the distribution of body sizes in cetacean populations have been interpreted as an indication of environmental changes (either a degradation [10] or an improvement [11] in prey availability), or as the direct consequence of anthropogenic stressors (e.g. whaling [9]). Bioenergetic models posit that absolute body size contributes to the success of a reproductive attempt [12,13], because larger females can carry more energy to invest in the calf and to buffer against environmental variation during reproduction (e.g. [14,15]).

Among baleen whales, a multi-decadal decline in mean asymptotic length was documented in North Atlantic right whales (*Eubalaena glacialis*; hereafter right whales) well after whaling was prohibited [16]. This species is critically endangered as a result of a complex set of stressors that both kill individuals and cause a progressive deterioration of their health [17,18]. Sublethal health effects have been associated with lower calving success [17,19], a delay in the age at first reproduction [20] and a reduction in annual calf production [21], resulting in low reproductive rates [22]. Combined with high levels of mortality and serious injuries from human activities, low reproductive rates likely contribute to the decreasing population trend [23,24]. Using an individual-based state-space model for health, survival and reproduction, Pirotta *et al*. [25] showed that female calving probability has been continuously declining over the last five decades. This model showed that calving probability was associated with the health metric, which also declined over time, but a large portion of the calving decline was ascribed to individual-level variability unrelated to health. Using a subset of females in the population, Stewart *et al*. [26] found a correlation between asymptotic body length and summary lifetime metrics of reproductive success (in particular, the number of births per reproductive year). Decreasing mean body size is therefore a good candidate trait for resolving the component of the decline in calving probability that was unrelated to concurrent health in the analysis of Pirotta *et al*. [25]. However, relying on lifetime reproductive metrics is limiting, as it constrains the analysis to whales that have reproduced and thus excludes many of the smallest individuals in the dataset that have yet to reproduce [26].

Here, we integrate the data and analysis of right whale length in the model for individual health and vital rates by Pirotta *et al*. [25], focusing on the temporal decline in mean asymptotic length rather than its causes. This integration examines a female's calving probability at each reproductive opportunity and thus (i) extends the analysis by Stewart *et al*. [26] to all females in the dataset (including unknown-age females and females that have not yet reproduced), (ii) quantifies the contribution of length to calving probability while accounting for variation due to health at each reproductive opportunity, and (iii) appropriately propagates the uncertainty across different data streams. Understanding what drives declining reproductive output in this species will be critical for the prioritization of conservation and management strategies to tackle the range of diverse stressors that threaten its viability [27,28]. Moreover, our analytical approach could be applied to investigate the effects of declining mean body size on vital rates across the range of long-lived species where a similar decreasing trend has been detected [29].

## 2. Methods

The analyses presented here build on the model described in Pirotta *et al*. [25]. This is a Bayesian state-space model for the survival and calving probability of individual right whales as a function of health status at a three-month scale. A set of intrinsic (lactation and juvenile status) and extrinsic (occurrence of vessel strike or entanglement, and a proxy for prey abundance) variables are modelled to affect latent health. The model is informed using 1970–2020 data accessed through the North Atlantic Right Whale Consortium (NARWC; www.narwc.org/narwc-databases.html), comprising individual sightings, health scores from a visual health assessment, information on sex, age class and calving, and records of deaths and anthropogenic traumas. Details of the model structure are provided in Pirotta *et al*. [25], while a summary of the data is reported in the electronic supplementary material.

In the original model by Pirotta *et al*. [25], calving probability (*φ_i,y_*) in year *y* when female *i* is available to give birth (i.e. alive, sexually mature, not in a pregnancy year, and not resting in a year after calving [19]) is related to health at some three-month time step *l* prior to *y* (*h_i,l_*) via a sigmoid function:

2.1 $$\varphi_{i,y}=g_{i}\left(\frac{m_{i}}{1+e^{-\delta (h_{i,l}-\mu )}}\right),$$

where *m_i_* is the asymptote of the sigmoid relationship representing the maximum calving probability for individual *i*; *δ* is the steepness of the sigmoid relationship; and *µ* is the value of health at which calving probability is 50% of the maximum. The binary variable *g_i_* indicates whether a female is reproductive (i.e. has already calved or may do so in the future), with mean probability *ν*. In Pirotta *et al*. [25], the individual-specific asymptote was modelled as an individual random effect around mean *λ* with standard deviation *χ*. Different lags between health and calving probability were tested, but they did not affect the estimated relationship. Therefore, health in the September–November interval, just prior to the breeding season, was used. Calving probability was related to observations of calving events:

2.2 $$r_{i,y}∼\mathrm{Bernoulli}(\varphi_{i,y}\;s_{i,y}),$$

where *r_i,y_* = 1 when the individual was seen with a calf on year *y*, 0 when it was sighted on that year but never with a calf, and unknown otherwise (and imputed in the Bayesian model); and *s_i,y_* = 1 when the individual was alive, and 0 otherwise.

The model was extended here to include a process component for individual length. Specifically, the length of individual *i* at each 3-month time step *t* (*L_i,t_*) was modelled following Stewart *et al*. [16] using a Gompertz growth function:

2.3 $$L_{i,t}=A_{i}e^{-Ce^{-Ka_{i,t}}},$$

where *C* regulates the position of the curve along the *x*-axis, *K* is the growth rate, and *a_i,t_* is individual age. This functional form was selected for consistency with Stewart *et al*. [16] and because it provided the best fit for North Atlantic right whales in Fortune *et al*. [30] (but see electronic supplementary material where we test the sensitivity of the results to this choice). Age was only known for a subset of individuals; see electronic supplementary material for details of the age prior for unknown-age individuals and for exploration of the effects of different assumptions surrounding sighting probability on the results. As in Stewart *et al*. [16], the individual-specific length asymptote *A_i_* was modelled as a function of an individual's birth year (*B_i_*):

2.4 $$A_{i}=A+\upsilon B_{i}+\varepsilon_{i},$$

where *A* is the intercept, *υ* is the effect of birth year and *ε_i_* is a normally distributed random effect with mean 0 and standard deviation *σ_A_*. Based on preliminary analysis of the data (see electronic supplementary material), *B_i_* was rescaled as max(*B_i_* – 1977, 0), so *B_i_* = 0 when an individual was born prior to or in 1977 (the first year where a birth event was recorded). Therefore, *A* represented mean asymptotic length for animals born until 1977.

We then modelled the effect of current length on calving probability in available years (*φ_i,y_*) by modifying the equation for the asymptote of the sigmoid function (now *m_i,y_*) for individual calving probability. Specifically, this was formulated using a logit transformation:

2.5 $$\mathrm{logit}(m_{i,y})=M_{i}+\zeta L_{i,l}^{3},$$

where $M_{i}∼\mathrm{Normal}(\lambda ,\chi )$ is the normally distributed, individual-specific intercept, and *ζ* is the effect of length cubed (using the same time step *l* prior to *y* as for the relationship with health). In the electronic supplementary material, we explore how this functional form compares to others at capturing the relationship between length and calving probability. We also conducted a preliminary exploration of the effect of length on survival probability but did not find support for its inclusion (electronic supplementary material).

Individual length and growth were informed using photogrammetric measurements derived from aerial images. Details of photogrammetry data collection are reported in Stewart *et al*. [16], and summarized in the electronic supplementary material. An observation model was included to describe measurement uncertainty, of the form

2.6 $$P_{i,t}∼\mathrm{Normal}(L_{i,t},\xi_{d}),$$

where *P_i,t_* is measured length for individual *i* in time step *t*, and *ξ_d_* is the observation error, specific to each methodology *d* used for measuring camera altitude (see electronic supplementary material).

Model fitting was undertaken using Markov chain Monte Carlo, implemented using the JAGS software [31] accessed via R [32]. Further details of model fitting, convergence and mixing diagnostics, and the prior distributions of all model parameters are provided in the electronic supplementary material. Analysis of variance was used to decompose the variation in predicted calving probability of reproductive females in each year into the contributions of length, health and individual random effect.

## 3. Results

On the basis of the diagnostics described in the electronic supplementary material, the model showed satisfactory mixing and convergence. Posterior estimates of all model parameters are reported in electronic supplementary material, table S1. Estimates reported here are posterior means followed by 95% equal-tailed credible intervals in square brackets.

**Figure 1.**
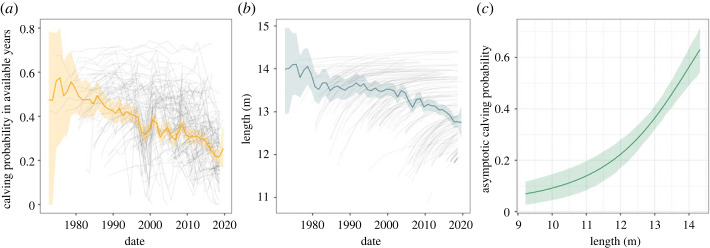
Individual calving probability in available years over the study period (*a*), length of reproductive females at reproductive opportunities (*b*) and estimated cubic effect on the asymptote of calving probability (*c*). In (*a*,*b*), each thin grey line corresponds to the time series for one female, while the thick coloured line and ribbon indicate the posterior mean and 95% credible interval across females. In (*c*), the green line and ribbon represent the posterior mean and 95% credible interval.

We found that the asymptotic length of individual right whales has declined since 1977 at a rate of −0.044 m yr^−1^ [−0.056 to −0.033] (figure 1*b*). In the electronic supplementary material, we show that the estimated magnitude of this decline was partly influenced by the limited number of length measurements for individuals born in the early years of the study period. In addition to the temporal trend, the length asymptote showed some residual individual-level variation, with standard deviation 0.5 m [0.4–0.6].

**Figure 2.**
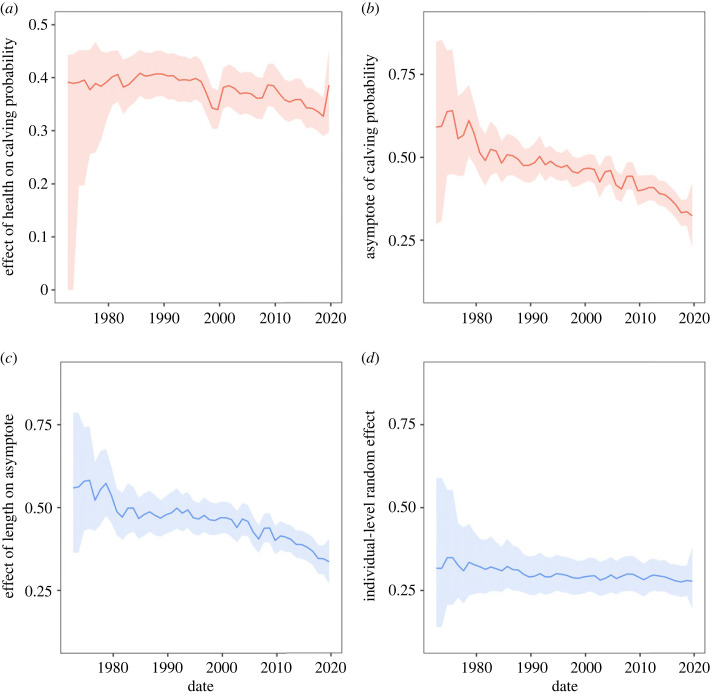
Estimated components of calving probability in available years over the study period. Top row: (*a*) mean contribution of health to calving probability, given mean length (13.3 m) and random effect equal to the mean (−0.86); (*b*) mean asymptote of calving probability across females. Bottom row: (*c*) mean contribution of length to the asymptote of calving probability, calculated by setting the intercept to the mean (−0.86); (*d*) mean individual random effect on the asymptote of calving probability. The lines and ribbons indicate the posterior means and 95% credible intervals in each year.

The cube of length was estimated to have a positive effect on calving probability (0.75 [0.52–1.00]; figure 1*c*), so that, on average, an 11 m long female was expected to have a 0.14 [0.09–0.20] maximum probability of giving birth to a calf on a year when she was available to do so, compared to a 0.56 [0.49–0.64] maximum probability for a 14 m long female. Mean calving probability was estimated to have declined consistently over the study period (figure 1*a*). When separating the different components of calving probability (figure 2), a large portion of its variation was captured by the trend in the calving probability asymptote (i.e. a female's maximum calving probability; figure 2*b*), while a smaller proportion was explained by changes in health (20% of variance in calving probability; figure 2*a*). The effect of length captured most of the decrease in the asymptote (figure 2*c*), explaining 62% of calving probability overall. By contrast, the contribution of the individual-level random effect was reduced (14% of total variance) compared with when the effect of length was not included (the standard deviation dropped from 0.80 [0.56–1.08] to 0.55 [0.23–0.83]), and it did not show a strong temporal trend (figure 2*d*).

## 4. Discussion

Our results provide strong evidence that the body size of a female right whale is closely associated with the probability of giving birth to a calf. The decreasing trend in individual asymptotic length [16] was able to capture most of the inter-individual variation in fecundity and explain the decline in calving probability over the last five decades [25]. Therefore, the reduction in length exacerbates the progressive deterioration in right whale health detected in our model and prevents females from reproducing more successfully even when their relative health is good because of lower absolute energy reserves [25], supporting the predictions from marine mammal bioenergetic models [12,13]. Age and experience could also play a role in the observed decline, although preliminary investigations do not support this hypothesis (electronic supplementary material).

We found that the relationship between the current length of a female and her calving probability is better represented by a cubic function (length^3^) than by length itself, which is in line with body volume (and therefore the total amount of reserves an individual carries given its structural size [5]) being the driver of a successful calving event. This relationship is formulated on the asymptote of the calving model, and thus affects the maximum reproductive probability of a female. At each putative calving event, the probability of achieving that maximum is then influenced by her current health status. In preliminary explorations, we investigated whether the model for calving probability could be formulated as a simpler logistic regression with both length and health as covariates. However, a mechanism is required to account for the lack of reproduction in some females in apparent good health and of sufficient length, which is likely related with other aspects of health or individual characteristics (e.g. fertility) that we do not measure, making asymptotic calving probability less than 1 [25].

Overall, body size thus acts as an integrator of individual health over time, because growth rate is affected by longer-term environmental conditions and exposure to anthropogenic stressors, compared to body condition and health [33,34]. In addition, the size an individual can achieve is likely influenced by its size at birth and weaning, which is associated with the genetic makeup of the mother as well as her health and body condition during gestation and lactation (e.g. [35]). Evidence for this exists in different baleen whale species, where bigger, fatter mothers produce larger calves [36,37], as well as in other mammals (e.g. [38]). Shorter calves might then not be able to fully compensate with increased growth even when conditions are good, leading to poorer life-history performance [39]. In right whales, it is unclear whether smaller individuals can make up for missed growth in the future. Stewart *et al*. [16] suggested that shorter right whale asymptotic length is associated with the duration of time an individual is entangled in fishing gear and with the entanglement status of its mother, but an in-depth analysis of the effect of multiple stressors and their interactions is needed, as well as a more direct quantification of total energy reserves (e.g. estimating body volume from photogrammetric width and length measurements). It should be noted that, despite the declining trend in mean body length, females born more recently (and characterized by shorter length at the putative age of maturity) did not appear to wait until they reached a longer body length before reproducing for the first time, as suggested by an exploration of body length at the first calving event (electronic supplementary material).

Our study adds to the empirical evidence and life-history theory indicating that, across species, changes in distribution of body sizes in a population affect reproductive success (e.g. in red squirrels *Sciurus vulgaris* [33], red deer *Cervus elaphus* [40], polar bears *Ursus maritimus* [34] and harbour porpoises *Phocoena phocoena* [11]), as well as being an important driver of several other life-history processes (e.g. thermoregulation and metabolic rates) [2]. The model we propose here provides a mechanistic approach to link health and body size with the probability of reproducing, which could be applied to quantify the contributions of different drivers of fecundity in other long-lived or iteroparous species where mean body size has been declining. Empirical evidence of the relationships between body size and vital rates is critical to forecast population trajectories and inform conservation strategies in a changing environment. This may particularly be the case for capital breeders, which rely on stored energy reserves to support reproduction [3]. If reduction in body size is confirmed as a common response to global climate changes [6], smaller size could have cascading effects on wildlife resilience to these changes and to other stressors, and indeed provide an early warning of population collapses [41].

North Atlantic right whales are critically endangered and declining [23]. The survival probability of individuals can be severely reduced by entanglements in fishing gear and vessel strikes [42,43]. In addition, their overall health status has been declining due to the sublethal effects of these and other anthropogenic stressors and changing environmental conditions [17,19,25], which is also leading to shifts in their distribution [44]. As a result, North Atlantic right whales are in poorer body condition compared with their southern congeners [45]. Decreasing mean body size and its effects on reproductive performance is another concerning indication of the progressive worsening of this species' prospects, requiring urgent interventions. While removing direct threats to survival is a first necessary step, management policies that enhance female health and growth to achieve higher rates of reproduction will ultimately be necessary to support the recovery of this and other populations affected by environmental change.

## Data Availability

The processed data and code used for the analyses are available from the Open Science Framework (https://osf.io/93znw/) [46]. The raw data are publicly available from the NARWC database collection (www.narwc.org/narwc-databases.html). Additional information is provided in electronic supplementary material [47].
